# Light-Dependent Activation of the GCN2 Kinase Under Cold and Salt Stress Is Mediated by the Photosynthetic Status of the Chloroplast

**DOI:** 10.3389/fpls.2020.00431

**Published:** 2020-04-29

**Authors:** Ansul Lokdarshi, Philip W. Morgan, Michelle Franks, Zoe Emert, Catherine Emanuel, Albrecht G. von Arnim

**Affiliations:** ^1^Department of Biochemistry & Cellular and Molecular Biology, The University of Tennessee, Knoxville, Knoxville, TN, United States; ^2^Department of Biology, Washington University in St. Louis, St. Louis, MO, United States; ^3^Graduate School of Genome Science and Technology, The University of Tennessee, Knoxville, Knoxville, TN, United States

**Keywords:** GCN2, eIF2α, cold, salt, light, translation, ROS

## Abstract

Regulation of cytosolic mRNA translation is a key node for rapid adaptation to environmental stress conditions. In yeast and animals, phosphorylation of the α-subunit of eukaryotic translation initiation factor eIF2 is the most thoroughly characterized event for regulating global translation under stress. In plants, the GCN2 kinase (*General Control Nonderepressible-2*) is the only known kinase for eIF2α. GCN2 is activated under a variety of stresses including reactive oxygen species (ROS). Here, we provide new evidence that the GCN2 kinase in Arabidopsis is also activated rapidly and in a light-dependent manner by cold and salt treatments. These treatments alone did not repress global mRNA ribosome loading in a major way. The activation of GCN2 was accompanied by a more oxidative environment and was attenuated by inhibitors of photosynthetic electron transport, suggesting that it is gated by the redox poise or the reactive oxygen status of the chloroplast. In keeping with these results, *gcn2* mutant seedlings were more sensitive than wild type to both cold and salt in a root elongation assay. These data suggest that cold and salt stress may both affect the status of the cytosolic translation apparatus via the conserved GCN2-eIF2α module. The potential role of the GCN2 kinase pathway in the global repression of translation under abiotic stress is discussed.

## Introduction

The translation of mRNAs by cytosolic ribosomes into new proteins is dynamically regulated by abiotic environmental conditions such as temperature (Matsuura et al., 2010; Yanguez et al., 2013), oxygen (Branco-Price et al., 2008), and light (Juntawong and Bailey-Serres, 2012; Liu et al., 2012; Missra et al., 2015; Merchante et al., 2017). Both early and more recent studies have highlighted that redox poise and reactive oxygen species (ROS) can also play important roles in regulating mRNA translation in global and mRNA sequence-specific ways (Tang et al., 2003; Branco-Price et al., 2008; Khandal et al., 2009; Juntawong and Bailey-Serres, 2012; Liu et al., 2012; Yanguez et al., 2013). The mechanisms that regulate and coordinate mRNA ribosome loading across the plant transcriptome are generally only partially understood. Of the several mechanisms regulating global translation, phosphorylation of the α-subunit of the heterotrimeric eukaryotic initiation factor 2 (eIF2) is one of the best characterized translational control events in yeast and animals (Dever et al., 1992; Sattlegger et al., 1998; Donnelly et al., 2013; Hinnebusch et al., 2016). In the unphosphorylated form, eIF2 bound to GTP delivers the initiator methionyl-tRNA to the small ribosomal subunit (40S) to initiate mRNA translation (Hinnebusch et al., 2016). Upon phosphorylation by one of several kinases, eIF2α then becomes a poisoned substrate of the guanine nucleotide exchange factor, eIF2B (Kashiwagi et al., 2019), causing global translational repression. Some mRNAs do escape this global repression by virtue of specific mRNA sequence elements (Harding et al., 2000; Liu and Qian, 2014).

General Control Non-derepressible 2 (GCN2) is the only known kinase in plants that phosphorylates eIF2α (Zhang et al., 2002; Lageix et al., 2008). In the well-studied vertebrate and yeast models, the GCN2 kinase can be activated by uncharged tRNA as a consequence of amino acid starvation (Wek et al., 1989, 1995; Dong et al., 2000; Anda et al., 2017). In plants, the genetic elements of the GCN2 pathway appear to be substantially conserved, although not all biochemical details have been confirmed, and few of the biochemical steps have been investigated thoroughly. Specifically, GCN2 is encoded by a single gene in Arabidopsis that functionally complements a yeast *gcn2* mutant (Zhang et al., 2003) and can be activated by uncharged tRNA *in vitro* (Li et al., 2013). Accordingly, *in planta*, the kinase is activated by inhibitors of amino acid biosynthesis such as the herbicides chlorosulfuron, glyphosate, and glufosinate (Lageix et al., 2008; Zhang et al., 2008; Zhao et al., 2018), and the activation of GCN2 by herbicides can be suppressed by supplementation with amino acids (Zhang et al., 2008).

Aside from inhibitors of amino acid biosynthesis, plant GCN2 kinase is activated by numerous other agents, including ultraviolet light, wounding, the ethylene precursor 1-aminocyclopropane carboxylic acid, the endogenous defense signals salicylic acid and methyl-jasmonate and bacterial infection (Lageix et al., 2008; Liu et al., 2019). What remains unclear is the nature of the biochemical signal that activates GCN2 under this variety of abiotic and biotic stresses. We recently described that GCN2 is activated by light-dependent ROS from the chloroplast. Even the stimulation of GCN2 by inhibitors of amino acid biosynthesis requires light and does not occur in darkness, suggesting that ROS are an essential requirement for GCN2 activation (Lokdarshi et al., 2020). A second conundrum surrounding plant GCN2 is that *gcn2* mutants have rather mild phenotypes under favorable lab conditions (Liu et al., 2015b) and a near-normal transcriptome (Faus et al., 2015; Lokdarshi et al., 2020). Moreover, among the various treatments that activate eIF2α phosphorylation, the herbicide chlorosulfuron is the only one that also results in a GCN2-dependent global translational repression (Lageix et al., 2008; Lokdarshi et al., 2020). In fact, the conditions that trigger eIF2α phosphorylation by the GCN2 kinase are not well correlated with the conditions under which *gcn2* mutant plants display maladaptive phenotypes.

Here, we describe that the GCN2 kinase is activated by cold and salt stress in a light-dependent manner. The activation of GCN2 by cold and salt can be suppressed by manipulating the status of the photosynthetic apparatus, suggesting that a chloroplastic signal contributes to the activation of GCN2. We also provide more evidence that eIF2α phosphorylation by different stresses does not always result in the same decline in polyribosome loading. However, *gcn2* mutant seedlings from two different ecotypes of Arabidopsis show reduced primary root growth under cold and salt stress, in keeping with a physiological role for the GCN2 kinase to adapt to these conditions. Taken together, these data suggest that the retrograde signaling from chloroplast to cytosol that targets protein synthesis may operate via the GCN2 kinase under cold and salt stress.

## Materials and Methods

### Plant Materials and Growth Conditions

*Arabidopsis thaliana* ecotype Landsberg (Ler-0), Columbia (Col-0), and homozygous *gcn2-1* mutants of the GT8359 gene trap line (Zhang et al., 2008) and homozygous *gcn2-2* (SALK_032196) mutant seeds (Faus et al., 2018; Lokdarshi et al., 2020) were sterilized and stratified at 4°C for 2 days. Seeds were germinated on half-strength Murashige-Skoog (1/2X MS) plant medium (MP Biomedicals, cat # 2633024) with 0.65% Phytoagar (Bioworld, cat # 40100072-2) and grown under a long-day period of 16 h light (80 ± 10 μEin m^–2^ s^–1^)/8 h dark at 22°C and 50% humidity. Unless stated, no sucrose was added to the medium.

### Stress Treatments and Phenotype Characterization

For cold stress treatment in dark and light, plates with 14-day-old horizontally grown seedlings (roots inside the medium) were acclimated in the dark for 24 h starting at Zeitgeber time 2 (ZT2), after which they were shifted to 4°C in the dark or light for the desired times. Dark-treated seedlings were harvested under green safe light. For salt stress treatment in the dark, plates with 9-day-old vertically grown seedlings (roots on the surface of the medium) were acclimated in darkness for 24 h starting at ZT2, after which seedlings were transferred to high salt or mock 1/2X MS salt media under green safe light, and sampling was performed at the desired times. For salt stress treatments under light, seedlings were germinated and grown vertically on agar medium supplemented with 0.1% sucrose for 10 days. At ZT2, seedlings were transferred quickly using a pair of tweezers to the same medium supplemented with high salt (150 mM NaCl), or control conditions, or control conditions with equivalent osmolarity of mannitol (300 mM).

For chemical treatments with DCMU (Thermo-Fisher, cat# D2425) and DBMIB (Thermo-Fisher, cat# 271993), seedlings were sprayed with the desired amount of reagent and mock control (DMSO or water) under green safe light 30 min before the end of 24 h dark acclimation. For antioxidant treatment, seedlings were germinated and grown for 10 days on 1/2X MS medium containing 0.5 mM ascorbate and 0.5 mM reduced glutathione.

For phenotype characterization under cold stress, 3-day-old vertically grown seedlings on 0.1% sucrose were transferred to media without sucrose and shifted to 4°C for 30 days. For salt stress, 3-day-old vertically grown seedlings on 0.1% sucrose were transferred to media with 0.1% sucrose (Mock) or supplemented with 300 mM mannitol or 150 mM NaCl. Photographs were taken with a digital camera (Canon) and primary root length was measured using ImageJ (ver. 1.4^1^). Fresh weight measurements were performed by weighing seedlings per plate at the end of the stress treatment. Percent survival analysis for salt stress was performed by counting seedlings that showed bleached chlorophyll and no primary root growth from days 6 to 9. All statistical analysis was performed using GraphPad Prism (ver. 8.1.2; GraphPad Software, Inc.).

### Protein Extraction and Immunoblot Analysis

Sampling for total protein extraction was done by flash freezing seedlings in liquid nitrogen. Seedlings were ground using a plastic pestle in a 1.5 ml tube with extraction buffer containing 25 mM Tris–HCl (pH 7.5), 75 mM NaCl, 5% (v/v) glycerol, 0.05% (v/v) Nonidet P-40, 0.5 mM EDTA, 0.5 mM EGTA, 2 mM DTT, 2% (w/v) insoluble PVP (Sigma P-6755), supplemented with 1 × protease and phosphatase inhibitor cocktail (Thermo-Fisher; cat# PIA32959). Total protein content was quantified by Bradford assay (Thermo-Fisher, cat# 23236).

For eIF2α phospho-immunoblot analysis, 50 μg of total protein was separated on a 12% (w/v) SDS-PAGE gel and electroblotted onto polyvinylidene fluoride (PVDF) membrane. After 1 h of blocking at 22°C with TBST buffer [1 × Tris-buffered saline (pH 7.6), 0.1% Tween-20] with 10% non-fat dry milk and 0.2% BSA, the membrane was washed 10 min each for 10 repeats and then incubated overnight at 4°C with rabbit polyclonal phospho-eIF2α antibody (Cell Signaling, cat # 9712S) diluted to 1:5000 in 1 × TBST with 0.5% BSA. Following washing with 1 × TBST, 10 min each for fifteen repeats, the membrane was incubated with horseradish peroxidase conjugated anti-rabbit IgG (Vector labs, cat# PI-1000) diluted to 1:2000 in 1 × TBST with 1% non-fat dry milk for 1 h at room temperature. After washing with 1 × TBST, 10 min each for 15 repeats, horseradish peroxidase was detected using chemiluminescence (WesternBright Quantum, Advansta) as per the manufacturer’s protocol. For immunoblot with rabbit polyclonal eIF2α antibody (a gift from Dr. Karen Browning, University of Texas, Austin), 5 μg of total protein was resolved by SDS-PAGE and electroblotted onto a polyvinylidene difluoride (PVDF) membrane. Blocking and incubation with antibodies were performed as previously described (Dennis et al., 2009) followed by chemiluminescent detection (Lokdarshi et al., 2016). Signal intensities on all immunoblots (Supplementary File S1) were quantified with ImageJ (ver. 1.41)^1^.

### Polysome Profiling and Protein Fractionation

Tissue for polysome profiling was harvested as described for total protein extraction. For polysome profiling with cold stress tissue, seedlings were ground in liquid N_2_ and 0.5 g of tissue powder was resuspended in 1 ml of polysome extraction buffer [200 mM Tris–HCl, pH 8.4, 50 mM KCl, 25 mM MgCl_2_, 1% deoxycholic acid, 2% polyoxyethylene-10-tridecyl ether, 50 μg/ml cycloheximide, and 40 U/ml RNase inhibitor (Promega Cat# N2115)] and centrifuged at 13,000 × *g* for 5 min at 4°C. One milliliter of the supernatant was layered onto a 10-ml 15–50% linear sucrose gradient prepared using a Hoefer gradient maker and centrifuged at 35,000 rpm (Beckmann SW 41 Ti) for 3.5 h at 4°C. Absorbance at 254 nm was recorded using an ISCO UA 5 absorbance/fluorescence monitor and individual data points were extracted using the DATA acquisition software (DATAQ instruments). Polysome-to-monosome (P/M) ratios were calculated as previously described (Enganti et al., 2018). For polysome profiling with salt stressed tissue, 150 mg of tissue powder was resuspended in 100 μl of polysome extraction buffer and centrifuged at 13,000 rpm for 5 min at 4°C. One hundred microliters of supernatant was layered on a 2-ml 15–50% linear gradient prepared as above and centrifuged at 50,000 rpm (Beckmann TLS55 rotor) for 1 h 10 min at 4°C. Absorbance was measured as described above.

### Hydrogen Peroxide Quantification

H_2_O_2_ content in seedlings was measured using the Amplex Red kit (Thermo-Fisher, cat# A22188). Briefly, 30 mg of 2-week-old seedlings were flash frozen in liquid N_2_ and ground with a plastic pestle to a homogeneous powder. Pulverized tissue was resuspended in 100 μl of sterile 1 × phosphate buffered saline (PBS) and centrifuged at 17,000 × *g* at 4°C for 2 min and the supernatant was used for H_2_O_2_ measurements as per the manufacturer’s protocol. Relative fluorescence was measured on a POLARstar OPTIMA plate reader (BMG LABTECH) with an excitation filter at 535 nm and emission filter at 600 nm.

### ROS Localization and Microscopic Techniques

Subcellular detection of ROS in Arabidopsis leaves was performed similar to Lokdarshi et al. (2020). Briefly, seedlings were submerged in 15 μM H_2_DCFDA (Thermo-Fisher, cat# D339) for 4–5 min in the dark. After rinsing seedlings twice with deionized water, ROS were imaged on a Leica SP8 laser scanning confocal microscope using the HeNe laser in the Advanced Microscopy and Imaging Facility at The University of Tennessee, Knoxville. The excitation filter was set to 488 nm and the emission filter was set to 500–550 nm for H_2_DCFDA and to 660–690 nm for chlorophyll autofluorescence. Confocal *z*-stack images were processed using ImageJ (ver. 1.4)^1^.

### Photosynthetic Efficiency Measurement

The maximum quantum yield of photosystem II (PS II) [Qymax = F_v_/F_m_] was measured on a FluorCam 800MF (Photon Systems Instruments) as per the manufacturer’s instructions and modifications from Murchie and Lawson (2013). Briefly, plants were dark adapted for 2 min (F_0_) prior to applying a saturating pulse of 1800 μEin m^–2^ s^–1^ for 0.8 s (F_m_). Variable fluorescence (F_v_) was calculated as the difference between F_o_ and F_m_ to get the maximum quantum yield [F_v_/F_m_]. For measurements under cold stress, pots with rosette stage wild-type and *gcn2* mutant plants on soil were shifted to cold (4°C) or left at 22°C (mock), and measurements were taken for the indicated times. Recovery from cold was done by moving the pot back to 22°C. For F_v_/F_m_ under salt stress, 3-day-old seedlings grown on 0.1% sucrose were shifted to 1/2X MS plant media supplemented with 150 mM NaCl or no salt as control (Mock) and F_v_/F_m_ measurements were recorded as discussed above.

## Results

### GCN2 Kinase Activation Under Cold Stress Is Light Dependent

**FIGURE 1:**
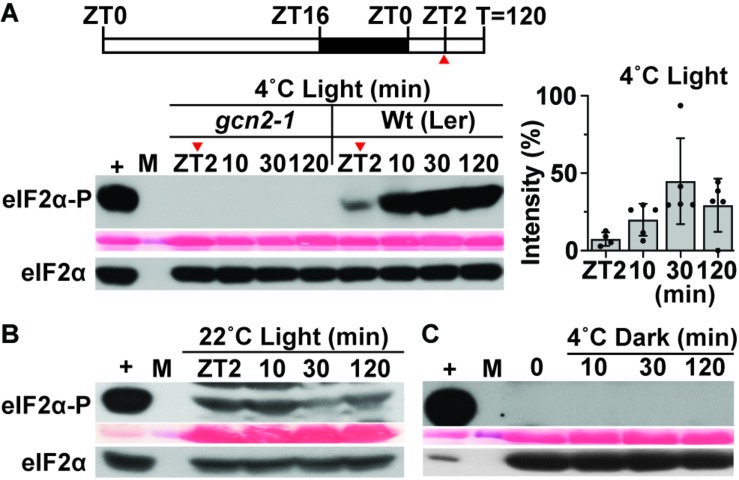
GCN2 kinase activation by cold is light dependent. **(A)** Top: Schematic of the light regimen. Wild-type Landsberg [Wt (Ler)] seedlings were grown for 14 days at 22°C in a 16 h light/8 h dark cycle and shifted to 4°C starting 2 h after lights-on [8 a.m., zeitgeber time (ZT)2]. The red arrow at ZT2 indicates the start of sampling right before the beginning of cold treatment. Bottom: Immunoblot showing the time course of eIF2α phosphorylation in 14-day-old Wt(Ler) and *gcn2-1* mutant (*gcn2-1*) seedlings subjected to cold stress as described in **(A)**. Upper panel: Probed with phospho-specific antibody against eIF2α-P (38 kDa). Middle panel: Rubisco large subunit (∼55 kDa) as a loading control after Ponceau S staining of the blot. Lower panel: Probed with antibody against eIF2α (38 kDa). (+), arbitrary amount of total protein extract from glyphosate treated Wt seedlings indicating unphosphorylated (eIF2α) or phosphorylated (eIF2α-P) protein; (10, 30, 120) sampling time in minutes; (M) Molecular weight marker. Also shown on the right is the variation in eIF2α-P levels (percent intensity) across the tested time periods in Wt seedlings. Error bars represent standard. deviation from five biological replicates. **(B)** Time course of eIF2α phosphorylation as in **(A)** but with Wt seedlings maintained at 22°C as a control. A cropped band at the top of the blot indicates non-specific binding of the antibody. **(C)** eIF2α phosphorylation in Wt seedlings under 4°C in the dark. Seedlings were grown in a 16 h light/8 h dark cycle, dark-acclimated for 24 h and shifted to 4°C in the dark. Time = 0 indicates the start of sampling in dark right before the cold treatment.

Previous reports (Lageix et al., 2008; Wang et al., 2017) showed eIF2α phosphorylation as a read out of GCN2 activity under cold stress. Given that the response to cold stress is closely linked to photosynthesis (Crosatti et al., 2013; Adam and Murthy, 2014; Zhu, 2016), we tested whether the activation of GCN2 under cold stress was light-dependent. In wild-type Arabidopsis seedlings subjected to 4°C cold in the light, phosphorylation of eIF2α increased gradually and remained high for at least 2 h of cold treatment. As expected, eIF2α phosphorylation was mediated by GCN2 (Figure 1A). In contrast, if the cold treatment was performed in dark-adapted plants, eIF2α remained unphosphorylated (Figure 1C). Under regular temperature conditions in the light, eIF2α-P remained steady between ZT2 and ZT4 (Figure 1B). Additionally, under all the test conditions, the overall amount of eIF2α remained unchanged (Figures 1A–C). These results show that GCN2-dependent eIF2α phosphorylation under cold stress is light dependent.

### Salt Stress Activates GCN2 in a Light Dependent Manner

**FIGURE 2:**
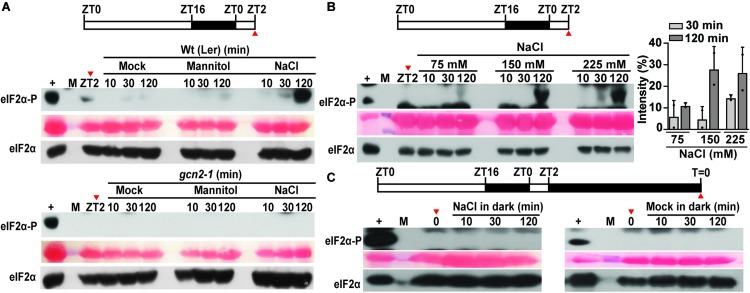
Salt stress activates GCN2 kinase in light. **(A)** Top: Schematic of the growth regimen. Wt seedlings were grown for 9 days at 22°C in a 16 h light and 8 h dark cycle. The red arrow at ZT2 indicates the start of sampling right before the beginning of salt stress treatment. Bottom: eIF2α phosphorylation in 10-day-old Wt (Ler) and *gcn2-1* mutant seedlings grown on medium containing 0.1% sucrose and shifted to mock conditions, or 300 mM mannitol or 150 mM NaCl. **(B)** Top: Schematics of the growth regimen. Bottom: eIF2α phosphorylation in 10-day-old Wt seedlings after shifting to different concentrations of NaCl (75, 150, 225 mM). Quantification of eIF2α phosphorylation at 30 and 120 min of NaCl treatment from two independent experiments is shown on the right. Error bars represent standard deviation. **(C)** Top: Schematics of 24 h dark acclimation starting at ZT2. Bottom: eIF2α phosphorylation in dark-acclimated Wt seedlings shifted to 150 mM NaCl (left) or to mock conditions (right). For details, see legend to Figure 1.

eIF2α has been shown to get phosphorylated in response to salt stress in mammals (Lu et al., 2001) and yeast (Goossens et al., 2001). To determine this response in plants, Arabidopsis seedlings grown in long-day period were shifted to 150 mM sodium chloride or an osmotically matched control (300 mM mannitol) (Figure 2A). Similar to other eukaryotes, salt treatment triggered eIF2α phosphorylation within 2 h only in the wild type but not in the *gcn2-1* mutant seedlings (Figure 2A). In addition, mock transfer and mannitol (osmotic control) did not activate GCN2. The increase in eIF2α-P was dosage dependent (Figure 2B). Similar to cold stress, salt stress has also been linked to adverse effects on chloroplasts in terms of photosynthesis and ROS accumulation (Parida and Das, 2005; Zhu, 2016; Suo et al., 2017; Robles and Quesada, 2019). To test the role of light under salt-triggered GCN2 activation, Arabidopsis seedlings were dark adapted for 24 h and shifted to salt or mock media. Salt treatment in the dark failed to activate GCN2 in wild-type seedlings, similar to the transfer control (Figure 2C). Taken together, both cold and salt stress require light to activate GCN2.

### Antioxidants and Photosynthetic Inhibitors Alleviate GCN2 Activity

**FIGURE 3:**
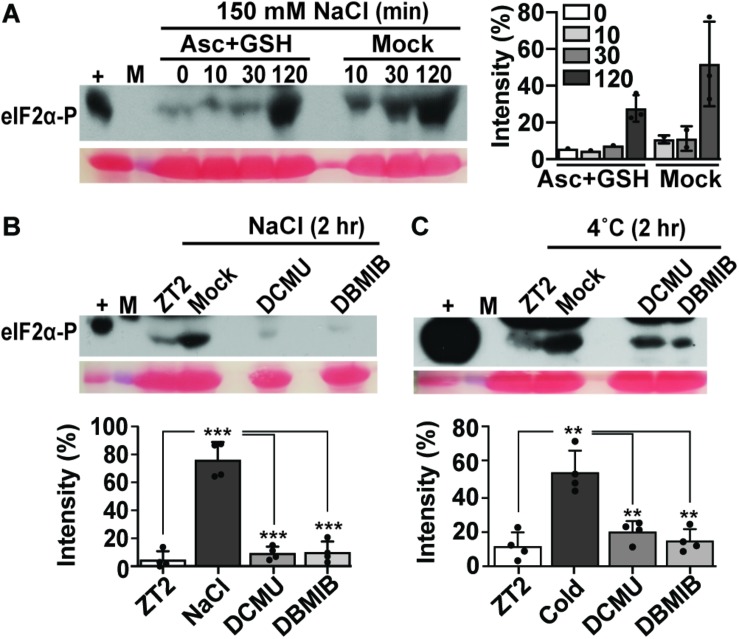
Antioxidant and photosynthetic inhibitors mitigate GCN2 kinase activation under cold and salt stress. **(A)** Time course of eIF2α phosphorylation in Wt seedlings grown on medium supplemented with 0.5 mM ascorbate and reduced glutathione for 10 days and shifted to 150 mM NaCl with either antioxidants (Asc + GSH) or mock control. Seedlings were transferred at ZT2 and harvested at 0, 10, 30, and 120 min. The graph shows eIF2α phosphorylation signals from three independent experiments with average, individual data points, and standard deviations. **(B,C)** eIF2α phosphorylation in Wt seedlings treated with either DMSO control (Mock), 8 μM of 3-(3,4-dichlorophenyl)-1,1-dimethylurea (DCMU), or 16 μM of 2,5-Dibromo-6-isopropyl-3-methyl-1,4-benzoquinone (DBMIB) 30 min prior to treatment for 2 h with **(B)** 150 mM NaCl or **(C)** 4°C cold. Welch’s unpaired *t-*test *P-*values for comparisons against NaCl/cold were *** < 0.001, ** < 0.01. For details see legend to Figures 1, 2.

In the light, low temperature and salt both affect PS II, resulting in an increase in the PS II excitation pressure, which generates damaging ROS, including hydrogen peroxide (Gray et al., 1996; Huner et al., 1998; Fowler and Thomashow, 2002; Murata et al., 2007). To address the role of photosynthetic electron transport for GCN2 activity, herbicides that manipulate the plastoquinone (PQ)/plastoquinol (PQH_2_) pool, 3-(3,4-dichlorophenyl)-1,1-dimethyl urea (DCMU), and 2,5-dibromo-3-methyl-6-isopropyl-p-benzoquinone (DBMIB) were applied shortly prior to the cold and salt treatments. DCMU keeps the PQ pool more oxidized (PQ) and DBMIB more reduced (PQH_2_) (Mateo et al., 2004; Kruk and Karpinski, 2006). Both herbicides suppressed salt- and cold stress-triggered GCN2 activation (Figures 3B,C). Prolonged cold and short salt stress (1 h or more) lead to ROS accumulation (Yao et al., 2018; Yuan et al., 2017). Here, we show that 2 h cold and salt treatments triggered mild but perceptible ROS accumulation in Arabidopsis seedlings, which was photosynthesis-dependent (Supplementary Figures 1A–D). To test whether ROS may contribute to GCN2 activation under cold and salt stress, seedlings were grown in the light on medium containing ascorbate and reduced glutathione before challenge with cold or salt stress. These antioxidants delayed the GCN2 activation, albeit weakly in the salt (Figure 3A) and not in the cold (not shown), possibly because antioxidants may be barely rate-limiting under these conditions. We acknowledge that the time course of eIF2α-P was faster than the apparent increase in ROS accumulation. That we failed to detect ROS sooner may be because ROS are an unstable and variable signal with a considerable basal level in the plant and often accumulate locally, while eIF2α-P is an endpoint signal that reports on an inherent signal amplification, the enzymatic kinase activity of GCN2. Taken together, these results, along with the light dependence of cold and salt stress on GCN2 activation, support the notion that chloroplast generated signals, possibly including ROS or redox signals, contribute to the activation of GCN2, leading to eIF2α phosphorylation.

### *gcn2* Mutant Sensitivity Toward Cold and Salt Stress

**FIGURE 4:**
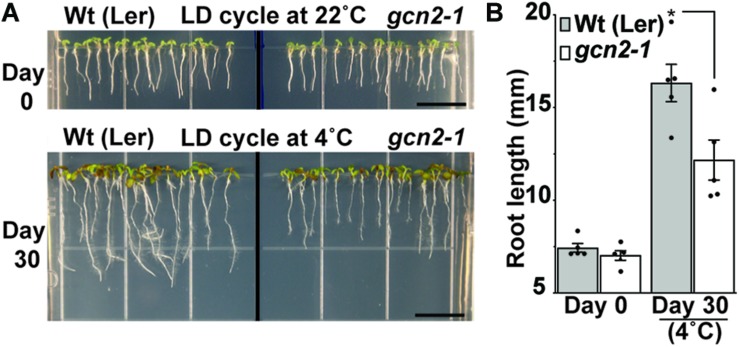
Loss of *GCN2* renders increased sensitivity toward cold stress. **(A)** Top: Representative images of 3-day-old Wt (Ler) and *gcn2-1* mutant seedlings grown under a 16 h light/8 h dark cycle (long day, LD) at 22°C. Seedlings were grown on medium with 0.1% sucrose for 3 days and transferred to no sucrose (day 0). Bottom: Same seedlings after 30 days of LD cycle at 4°C. Scale bars are 10 mm. **(B)** Primary root length of Wt and *gcn2-*1 mutant seedlings from **(A)**. Error bars indicate standard error of the mean from four biological replicates with *n* > 80 per experiment (Welch’s *t-*test *P < 0.05).

To determine the role of GCN2 specifically under cold and salt stress conditions at the whole plant level, an established *GCN2* mutant allele (*gcn2-1*) (Lageix et al., 2008; Zhang et al., 2008) in the Landsberg ecotype and a homozygous T-DNA insertion allele of *GCN2* in the Columbia ecotype (*gcn2-2)* (Faus et al., 2018) were tested for phenotypic abnormalities. Under normal growth conditions, *gcn2-1* mutants were indistinguishable from wild type in terms of both shoot and primary root growth (Figures 4A,B). However, after challenge with cold stress, *gcn2-1* mutant root lengths were retarded compared to wild type (Figures 4A,B) as were *gcn2-2* mutants (Supplementary Figures 2A,B). Of note, the defect in overall growth in the *gcn2* mutants could not be attributed to any defects in the photosynthetic quantum efficiency (Supplementary Figures 3A,B).

**FIGURE 5:**
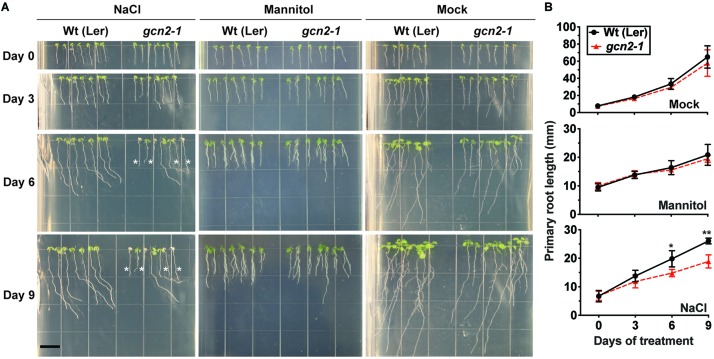
*gcn2* mutants are more sensitive to salt stress. **(A)** Wt (Ler) and *gcn2-1* mutant seedlings were grown under a 16 h light/8 h dark cycle for 3 days on plant medium supplemented with 0.1% sucrose. On day 3, seedlings were transferred to new plates with 0.1% sucrose and 150 mM NaCl (salt treatment), 300 mM mannitol (osmotic control), or no supplement (mock). Root growth was recorded for another 9 days. Scale bar is 10 mm. Seedlings that bleached out and died are indicated by asterisks. **(B)** Primary root length of Wt and *gcn2-1* mutants from **(A)**, excluding dead seedlings. Error bars indicate standard error of the mean of four biological replicates with *n* > 36 per experiment (Welch’s *t-*test *P < 0.05; **P < 0.005).

**FIGURE 6:**
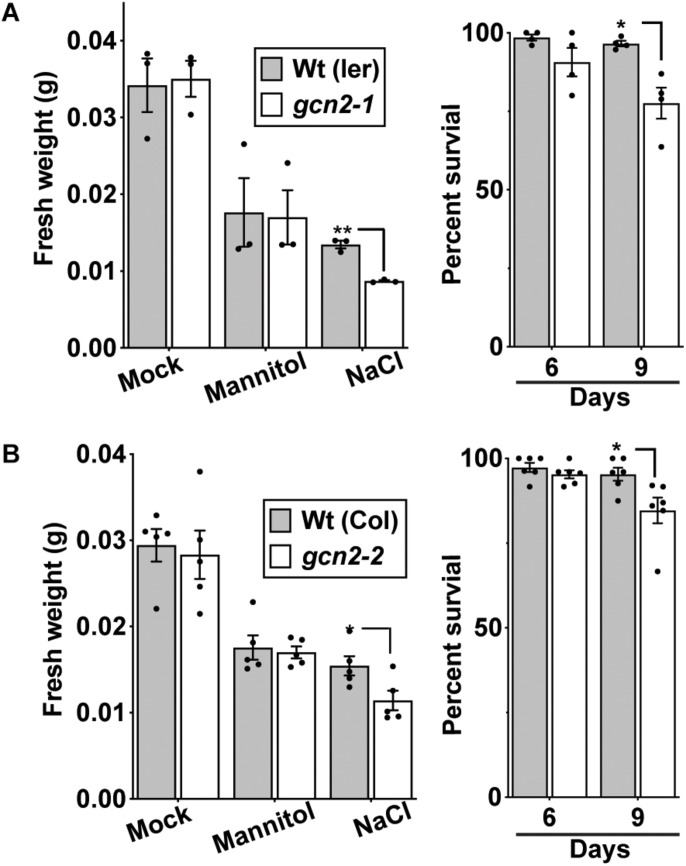
*gcn2* mutants accumulate less fresh weight and exhibit low survival under salt stress. **(A)** Left panel: Fresh weight (grams) of Wt(Ler) and *gcn2-1* mutant seedlings after 9 days of growth on 300 mM mannitol or 150 mM NaCl, or no supplement (Sucrose). The medium for all seedlings contained 0.1% sucrose. Right panel: Percent survival of Wt and *gcn2-1* mutant seedlings at days 6 and 9 on 150 mM NaCl. Data include the experiment in Figure 5. **(B)** Fresh weight and percent survival of wild-type Columbia [Wt (Col)] and *gcn2-2* mutant (*gcn2-2*) seedlings at days 6 and 9 on 150 mM NaCl. Data include the experiment in Supplementary Figure 4. Error bars indicate standard error of the mean of four biological replicates with *n* > 36 per experiment (Welch’s *t*-test *P < 0.05; **P < 0.005).

Similar to the root growth retardation in the cold, exposure of seedlings to 150 mM NaCl salt also retarded primary root growth in the *gcn2* mutants (Figures 5A,B and Supplementary Figures 4A,B). Additionally, some *gcn2* mutants developed extreme chlorosis and root growth arrest by days 6 and 9 (Figure 5A and Supplementary Figure 4A: denoted by asterisks). These effects were specific to salt and not seen in the osmotic control (mannitol) and transfer control (mock) treatments. The growth defect of the *gcn2* mutant on salt was evident by day 6 and resulted in a significant loss of fresh weight and percent survival by day 9 (Figures 6A,B). As previously seen for cold stress, the quantum efficiency of PS II declined similarly for *gcn2* and wild type under salt stress (Supplementary Figure 5). In these experiments, we noticed that the *gcn2* mutant strains have an increased probability as compared to wild type to assume a state of virtual root growth arrest, an effect that was particularly pronounced in the Col ecotype (Supplementary Figures 2, 4). We conclude that the GCN2 promotes adaptation of seedlings to cold and salt stress, specifically by increasing the probability that the seedlings can maintain root growth homeostasis under stress challenge.

### mRNA-Ribosome Loading Under Cold and Salt Stress

**FIGURE 7:**
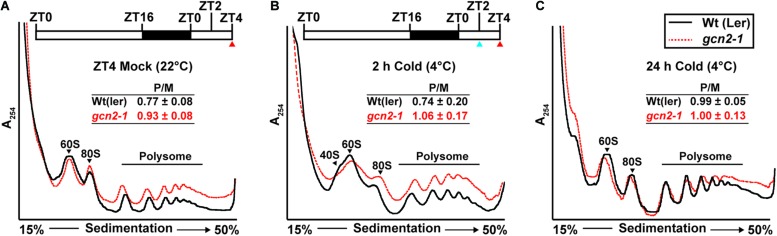
Ribosome-RNA profile of wild-type and *gcn2-1* under standard growth conditions and cold stress. Top: **(A,B)** Schematic of light regimen showing seedling growth in long-day period (16 h light and 8 h dark) indicating the beginning of cold (4°C) treatment starting at ZT2 (blue arrow) and the sampling time at ZT4 (red arrow). Bottom: UV absorbance profile at 254 nm of 14-day-old wild-type Landsberg [Wt(ler)] and *gcn2-1* mutant (*gcn2-1*) seedlings at **(A)** 22°C at ZT4 (Mock) or subjected to cold at 4°C **(B)** for 2 h, or **(C)** for 24 h under a long-day period. The positions of the 40S, 60S, 80S, and the polysomes are indicated on the profiles. The ratio of polysomes (P) to monosomes (M) is indicated with standard error from three replicates.

**FIGURE 8:**
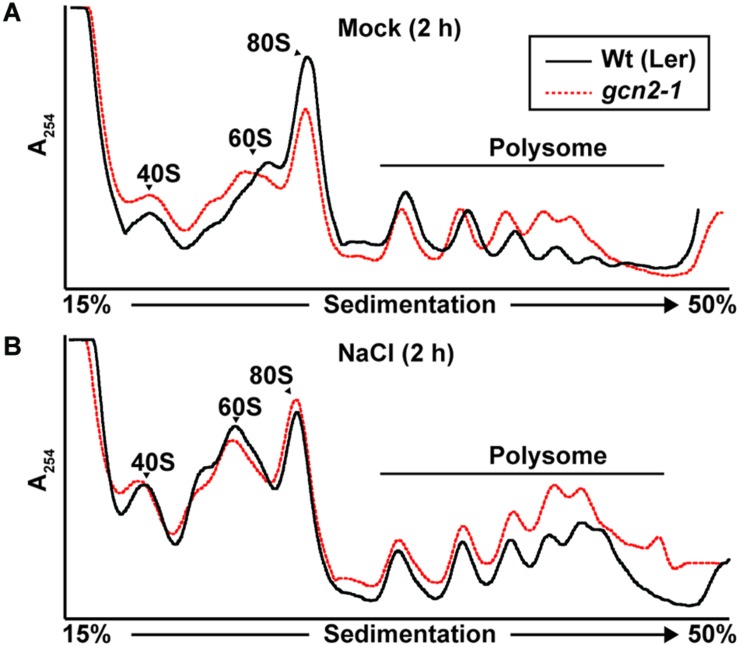
Ribosome-RNA profile of wild-type and *gcn2* mutant under salt stress. Representative UV absorbance (A254 nm) profile of 10-day-old Wild-type Landsberg [Wt(Ler)] and *gcn2-1* mutant (*gcn2-1*) seedlings after 2 h of treatment with **(A)** 0.1% sucrose (Mock) or **(B)** NaCl. Seedling transfer was performed as described in Figure 2. Positions of the 40S, 60S, 80S, and the polysome are indicated on the profile.

GCN2 activity has been implicated in the down-regulation of mRNA translation under a variety of stress conditions (Lageix et al., 2008; Zhang et al., 2008; Liu et al., 2015a; Wang et al., 2017; Llabata et al., 2019). To test the role of GCN2 in global mRNA translation under cold and salt stress, *gcn2* mutant and wild-type seedlings were challenged with the respective stresses under light. Polysome profiles from sucrose density gradients revealed overall similar profiles and polysome-to-monosome ratios for wild-type and *gcn2* under both normal growth conditions (Figure 7A) and after cold stress (Figures 7B,C). Likewise, in response to salt stress, both wild-type and *gcn2* mutant displayed similar polysome profiles (Figure 8). The trend toward slightly elevated ribosome loading in *gcn2-1*, while not uncommon, was not statistically significant. The lack of a clear effect on global polyribosome loading stands in contrast to data after herbicide treatment where ribosome loading declines in a GCN2-dependent manner (Lageix et al., 2008; Lokdarshi et al., 2020). Thus, with cold and salt, we have identified stress conditions that trigger eIF2α phosphorylation without causing transcriptome-wide translational repression across the entire plant.

## Discussion

The GCN2-eIF2α module is an integral component of a pan-eukaryotic stress response program. In yeast and mammals, GCN2 is activated by binding to uncharged tRNAs via its C-terminal HisRS domain. In plants, GCN2 kinase is activated under a wide range of abiotic stresses (e.g., UV light, cold, wounding), synthetic agents (e.g., herbicides, purine starvation), hormones (e.g., methyl jasmonate, salicylic acid, abscisic acid), and live bacterial pathogen (e.g., *Pseudomonas syringae*). More recently, Arabidopsis GCN2 was found to be activated in response to H_2_O_2_ directly, as well as excess light stress and methyl viologen, treatments that produce ROS (Lokdarshi et al., 2020). In the present study, we show that both cold and salt challenge not only activate eIF2α-P but require light to do so, similar to our recent findings of GCN2 activation in response to herbicide. Taken together, our study suggests that the highly conserved GCN2-eIF2α module is activated in a common manner by different stresses, possibly by ROS, given that H_2_O_2_ is the only known signal to activate GCN2 in darkness (Lokdarshi et al., 2020). The precise biochemical mechanism remains to be determined.

Biochemically, the only known ligand to activate plant GCN2 *in vitro* are uncharged tRNAs, which presumably accumulate in the cell during amino acid starvation. Whether uncharged tRNAs are necessary and sufficient to activate GCN2 *in planta* under all stress conditions remains unclear. It is plausible that tRNA is bound to GCN2 as a coactivator but that additional signals are needed to boost kinase activity to physiologically relevant levels. Of note, recently, Inglis and coworkers reported that mammalian GCN2 can be activated in a tRNA-independent mechanism by the ribosomal P-stalk protein complex (Sattlegger and Hinnebusch, 2000; Inglis et al., 2019). The mechanism of how GCN2 is activated *in planta* by tRNAs and ROS may also depend on the GCN2 interacting proteins GCN1 and GCN20 (Wang et al., 2017; Faus et al., 2018; Izquierdo et al., 2018), similar to yeast and mammals; however, plastidic ROS as a GCN2 activation signal is unique to plants.

It remains unclear whether and how the GCN2-mediated phosphorylation of eIF2α under various conditions drives global translational repression as seen at the level of polyribosome loading, and how this response supports plant growth and development. The clearest causal chain of events is observed with herbicides that inhibit amino acid synthesis, where activation of GCN2 kinase by herbicide in the presence of light-conditioned ROS causes eIF2α phosphorylation, followed by global translational repression, which is disrupted in the *gcn2* mutant (Lageix et al., 2008; Lokdarshi et al., 2020). Moreover, the *gcn2* mutant is hypersensitive to herbicide (Zhang et al., 2008; Izquierdo et al., 2018), all in keeping with a simple, linear signaling pathway. However, it is much less clear how other GCN2-targeted abiotic stimuli affect translation, notwithstanding that it has been confirmed multiple times that eIF2α phosphorylation is always mediated by GCN2. Here, we showed that upon cold treatment, eIF2α became phosphorylated by GCN2, but with no detectable translational repression by either cold or GCN2 kinase, although *gcn2* mutants were cold sensitive. We observed the same result for salt stress. Of note, salt stress at slightly higher intensity in rice (Ueda et al., 2012), but not cold stress in Arabidopsis (Juntawong et al., 2013), cause a drop in global ribosome loading. As for ROS, which we consider the most immediate activator of the GCN2 kinase, this stress represses translation as well as plant growth, but neither is detectably GCN2-dependent (Lokdarshi et al., 2020). The same pattern was seen in response to DTT and antimycin A (Izquierdo et al., 2018). Under high light, which is likely another relevant trigger of GCN2 in the natural environment, again, there is no GCN2-dependent translational repression, although *gcn2* mutants are sensitive to high light (Lokdarshi et al., 2020). For comparison, heat and hypoxia both rapidly repress global translation (Branco-Price et al., 2008; Matsuura et al., 2010; Yanguez et al., 2013), but without any apparent phosphorylation of eIF2α. Taken together, these observations clearly suggest that, despite the seemingly simple sequence of events in response to certain inhibitors of amino acid synthesis, not every instance of eIF2α phosphorylation causes global translational repression, and only some but not all instances of global translational repression are conditioned on eIF2α phosphorylation. These observations indicate that there must be additional translational control pathways that cooperate with GCN2-mediated eIF2α phosphorylation to organize the translatome under abiotic stress. Candidates are GCN1/ILITHYIA (ILA) (Wang et al., 2017) and GCN20-mediated (Izquierdo et al., 2018), autophagy-mediated processes (Yoon and Chung, 2019), processes involving SnRK-TOR signaling (Margalha et al., 2019), and stress granules (Chantarachot and Bailey-Serres, 2018). This conclusion is also in keeping with the emerging role of GCN2 in responses to plant pathogens. Under certain conditions, pathogens or effectors of immunity activate GCN2 or eIF2α phosphorylation (Liu et al., 2015b), while in other conditions, they do not (Zhang et al., 2008; Meteignier et al., 2017; Izquierdo et al., 2018). Certain pathogens do trigger translational reorganizations (Moeller et al., 2012; Xu et al., 2017) and GCN2 is involved in responses to bacterial pathogens (Liu et al., 2019; Lokdarshi et al., 2020) although the precise role of GCN2 kinase signaling in defense-related translational control remains to be defined.

Overall, the findings presented in this study add to a unified model of the regulation of the cytosolic translation apparatus via the highly conserved GCN2-eIF2α module under a variety of abiotic stresses that may also extend to biotic stresses in plants. In summary, we show that activation of GCN2 by cold and salt stress is dependent on the redox state of the chloroplast, and loss of *GCN2* results in the increased sensitivity toward common abiotic stress inputs, cold and salt. In the future, determining what biochemical and molecular events lead to GCN2 activation under these natural stress inputs will shed light on the integrated stress response pathway in plants. Additionally, the regulation of global translation versus specific mRNAs that fall under stress type regulation is also a subject of further investigation.

## Author Contributions

AL, PM, MF, ZE, and CE performed the experiments. AL and AA analyzed the results and wrote the manuscript.

## Conflict of Interest

The authors declare that the research was conducted in the absence of any commercial or financial relationships that could be construed as a potential conflict of interest.

## Footnotes

1 http://rsb.info.nih.gov/ij/index.html

## Supplementary Material

FILE S1 Original blots.
