# Supplementary material for: Light-Dependent Activation of the GCN2 Kinase Under Cold and Salt Stress Is Mediated by the Photosynthetic Status of the Chloroplast
Source: Front Plant Sci. 2020 Apr 29;11:431. doi: 10.3389/fpls.2020.00431 (PMC7201089; doi:10.3389/fpls.2020.00431)
Supplement: FILE S1 — Original blots. [file Data_Sheet_1.pdf]

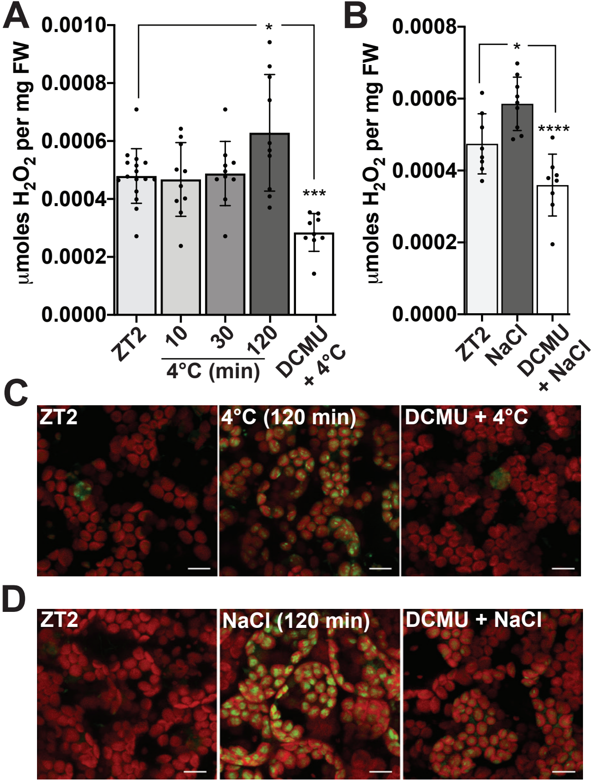

Supplemental Figure 1. Cold and salt treatments modulate hydrogen peroxide levels.
(A, B) Amplex red assay showing relative H_2O_2 levels in wild-type Landsberg (Wt) seedlings. (A) Cold and (B) Salt stress (120 min) treatments were performed as described in Fig. 1A and Fig. 2A respectively. Treatment with DCMU was performed as described in Fig. 3B,C and sampling was performed after 120 min of stress treatments. Error bars represent standard deviation of at least 7 biological replicates. Welch's t-test P-value *<0.05, ***<0.0005 , ****<0.0001.
(C, D) Representative images of Wt seedling leaves stained with the ROS sensitive dye H_2DCFDA after 120 min of (C) cold and (D) salt stress treatments as descibed in Fig. 1A, B respectively. Shown are the superimposed images with oxidized H_2DCFDA represented in green and chlorophyll in red. Scale bars are 10 µm.

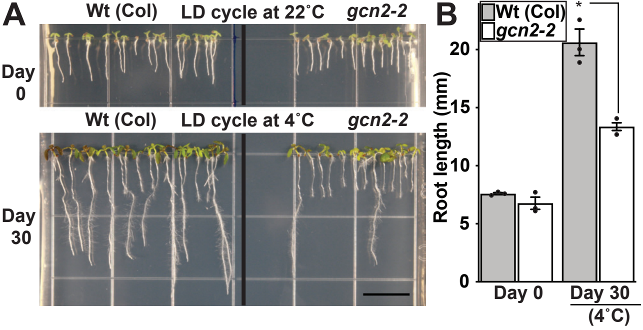

Supplemental Figure 2. Loss of *GCN2* renders increased sensitivity towards cold stress in the Columbia ecotype.
(A) Top - Representative images of 3-days-old wild-type Columbia (Wt (Col)) and *gcn2-2* mutant (*gcn2-2*) seedlings grown under 16 h light and 8 h dark long day (LD) cycle at 22°C. Seedlings were grown on media with 0.1% sucrose for 3-days and transferred to no sucrose (Day 0).
Bottom - Same seedlings after 30 days of LD cycle at 4°C. Scale bars are 10 mm.
(B) Primary root length of Wt and *gcn2-2* mutant seedlings from panel A. Error bars indicate standard error of the mean from four biological replicates with n>80 per experiment. (Welch's t-test *P-value <0.05)

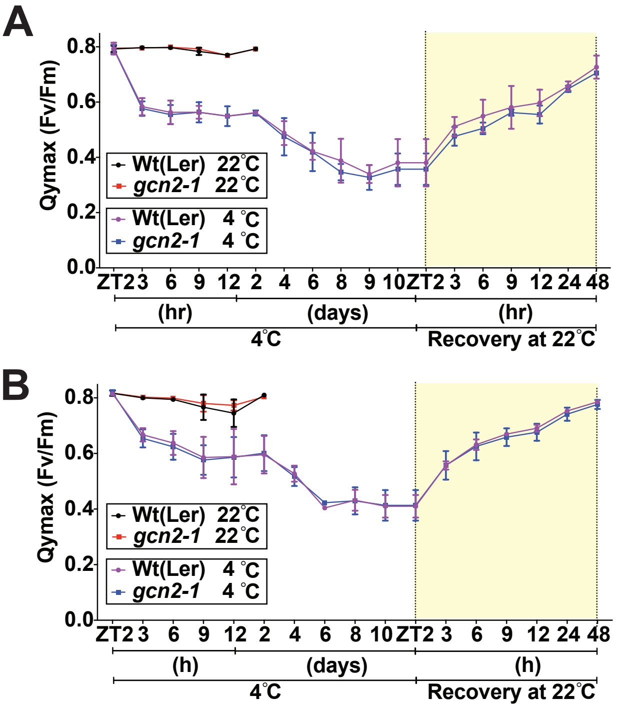

Supplemental Figure 3 . Effect of cold stress on photosynthetic efficiency of wild-type and *gcn2* mutants. Time course analysis of photosystem II maximal quantum yield (Qymax (Fv/Fm)) in rosette stage plants of **(A)** wild-type Landsberg (Wt (Ler)) and *gcn2-1* mutant , **(B)** wild-type Columbia (Wt(Col)) and *gcn2-2* mutants under cold stress at 4°C and recovery at 22°C. Error bars represent standard error of the mean from three biological replicates. Welch's t-test P-value >0.5.

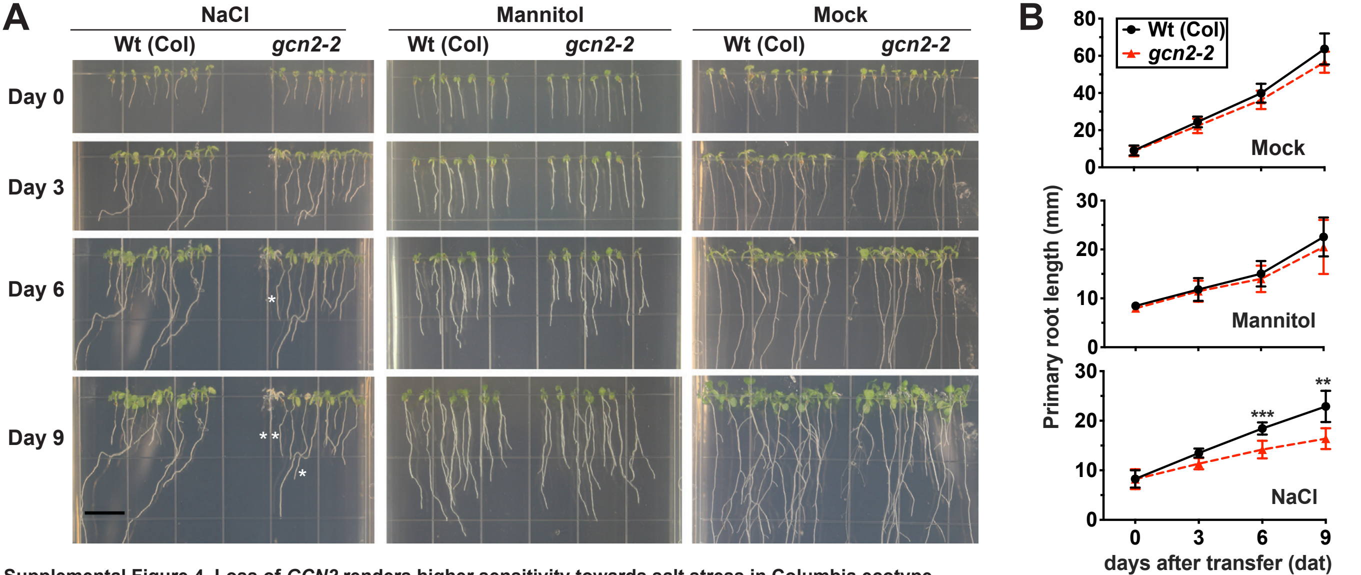

Supplemental Figure 4. Loss of *GCN2* renders higher sensitivity towards salt stress in Columbia ecotype

(A) Representative images of wild-type Columbia (Wt (Col)) and *gcn2-2* mutant (*gcn2-2*) seedlings grown under 16 h light and 8 h dark period. After 3-days on 0.1% sucrose plant media, seedlings were transferred to a fresh plate (Day 0) on the same medium (Mock), or supplemented with 150mM NaCl (salt treatment), or 300mM mannitol (osmotic control). Scale bar is 10mm. Bleached seedlings are marked with an asterisk and were excluded from root length measurements.

(B) Primary root length of Wt and *gcn2-2* mutants from panel (A). Error bars indicate standard error of the mean of four biological replicates with n>36 per experiment (Welch's t-test ** P-value <0.005; *** P-value <0.0005).

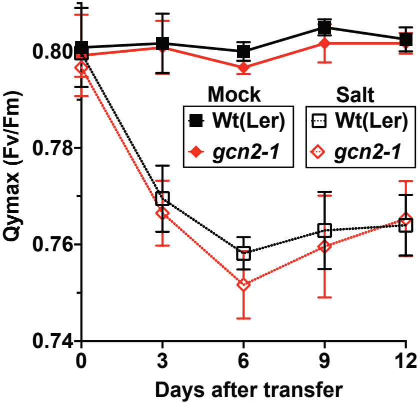

Supplemental Figure 5 . Effect of salt stress on photosynthetic efficiency of wild-type and *gcn2*. Time course analysis (Day) of PSII maximal quantum yield (Qymax (Fv/Fm)) of wild-type Landsberg (Wt (Ler)) and *gcn2-1* mutant *(gcn2-1)* seedlings after transfer to 0.1% sucrose (Mock) or 150mM NaCl (Salt) containing media. Error bars represent standard error of the mean from five biological replicates. Welch's t-test *P*-value >0.5.
