# Supplementary material for: Light-Dependent Activation of the GCN2 Kinase Under Cold and Salt Stress Is Mediated by the Photosynthetic Status of the Chloroplast
Source: Front Plant Sci. 2020 Apr 29;11:431. doi: 10.3389/fpls.2020.00431 (PMC7201089; doi:10.3389/fpls.2020.00431)
Supplement: Supplementary file 2 [file Data_Sheet_2.pdf]

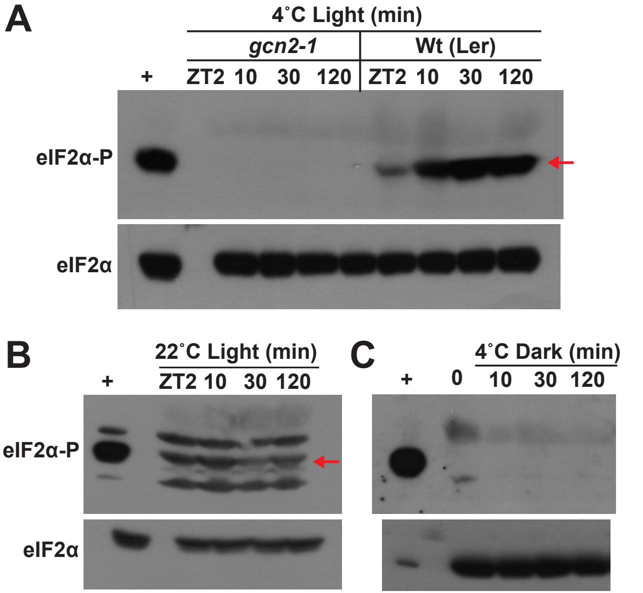

Original images of the blots supporting Figure 1. Please refer to the main text for explanation. Red arrow indicates the band for phosphorylated eIF2α.

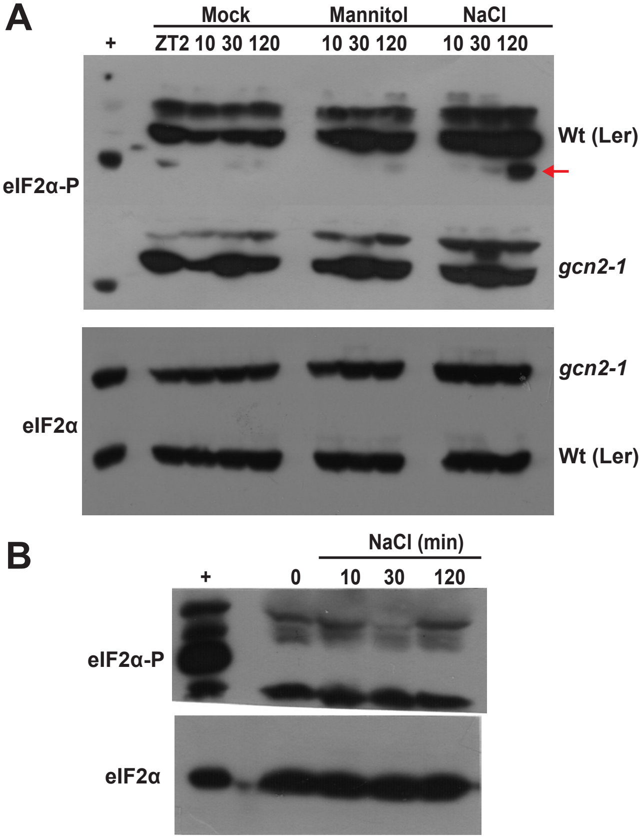

Original images of the blots supporting Figure 2. Please refer to the main text for explanation. Red arrow indicates the band for phosphorylated eIF2α.

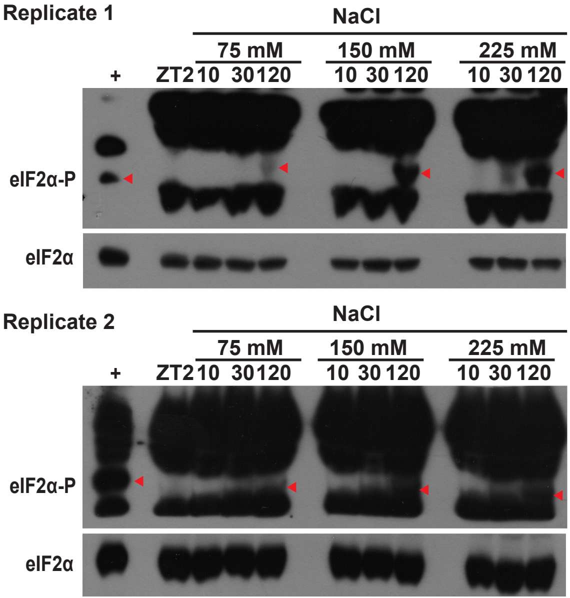

Original images of the blots supporting Figure 2B. Please refer to the main text for explanation. Red arrows indicate the band for phosphorylated eIF2α (eIF2α-P). Blots for unphosphorylated eIF2α are shown below respective eIF2α-P blots.

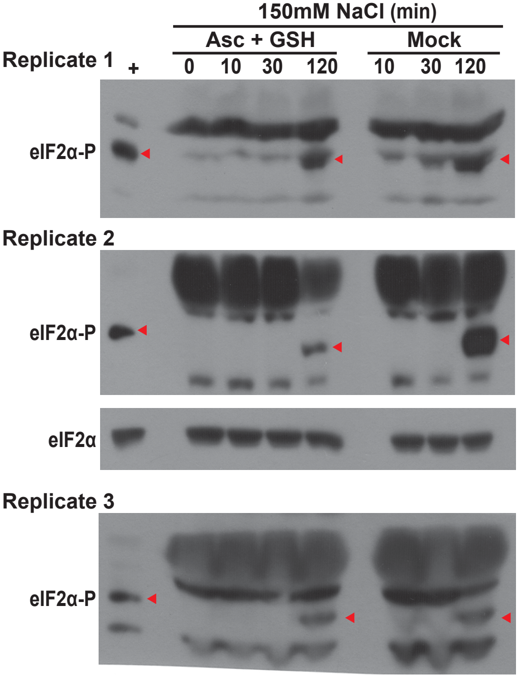

Original images of the immunoblots supporting Figure 3A. Please refer to the main text for explanation. Red arrows indicate the band for phosphorylated eIF2α (eIF2α-P). Immunoblot for unphosphorylated eIF2α is shown below eIF2 α-P immunoblot for replicate 2.

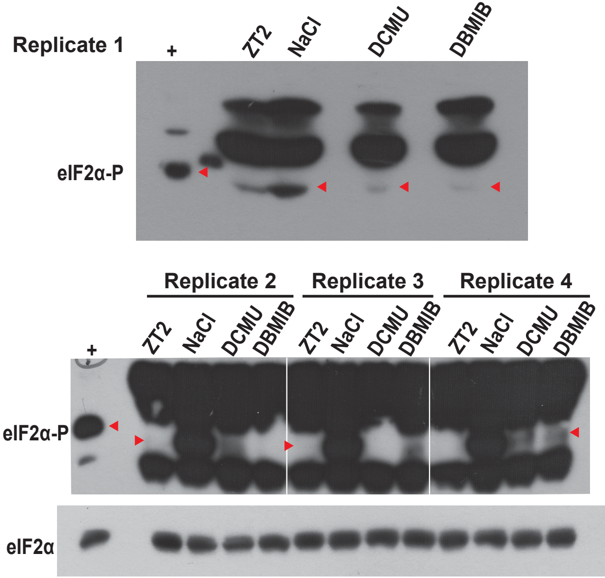

Original images of the immunoblots supporting Figure 3B. Please refer to the main text for explanation. Red arrows indicate the band for phosphorylated eIF2α (eIF2α-P). Immunoblot for unphosphorylated eIF2α is shown below eIF2 α-P blot for replicate 2,3 and 4.

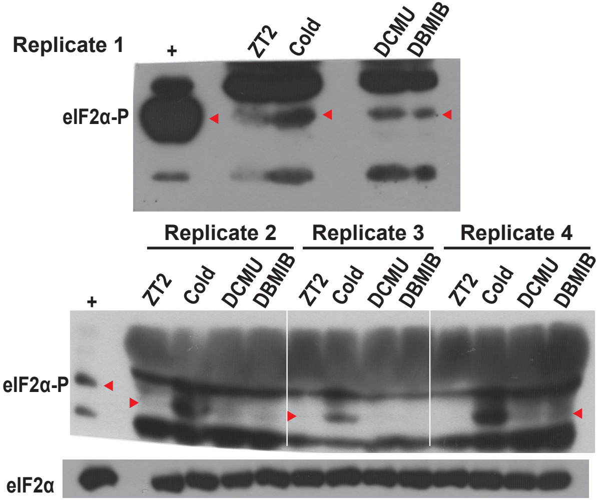

Original images of the immunoblots supporting Figure 3C. Please refer to the main text for explanation. Red arrows indicate the band for phosphorylated eIF2α (-P). Immunoblot for unphosphorylated eIF2α is shown below eIF2α-P blot for replicate 2,3 and 4.
